# Palatal erosion and oronasal fistulation following covered nasopharyngeal stent placement in two dogs

**DOI:** 10.1186/2046-0481-66-8

**Published:** 2013-05-01

**Authors:** Audrey K Cook, Kelley Thieman Mankin, Ashley B Saunders, Carly E Waugh, Laura C Cuddy, Gary W Ellison

**Affiliations:** 1 Department of Small Animal Clinical Sciences, College of Veterinary Medicine and Biomedical Sciences, Texas A&M University, College Station, TX 77843, USA; 2 Department of Small Animal Clinical Sciences, College of Veterinary Medicine, University of Florida, Gainesville, FL 32608, USA

**Keywords:** Canine, Palate, Nasopharyngeal stenosis, Stent

## Abstract

Treatment options for dogs with nasopharyngeal stenosis include fluoroscopic placement of metallic stents. Reported complications include entrapment of hair and food, obstruction and persistent nasal discharge. Two toy breed dogs were examined for persistent nasal discharge and halitosis at 4 and 20 months after placement of permanent metallic stents for acquired nasopharyngeal stenosis. Full thickness defects were found in the palate of both dogs, with extensive communication between the mouth and the nasal passages. Portions of the metal stent were observed within the lesion in both patients. Additional treatment was declined by the owner of one dog; the stent was removed through the fistula in the other dog. Palatal erosion with secondary oronasal fistulation is a potential complication of nasopharyngeal stent placement in dogs.

## Background

Nasopharyngeal stenosis secondary to regurgitation of gastric contents during anesthesia has rarely been reported in dogs [1,2]. Clinical signs associated with stenosis of the nasopharynx can be severe and the quality of life for affected animals is substantially compromised. Historically, attempts to alleviate this condition with surgery, endoscopic ballooning or laser ablation have been variably successful, and may in fact trigger progression from partial stenosis to complete occlusion [1,3-5].

In recent years, excellent short and long term outcomes have been reported with the use of metal stents for both dogs and cats with nasopharyngeal stenosis [1]. Patients with complete obstruction of the affected area appear predisposed to ingrowth through an open stent, so covered stents are recommended for these individuals.

Complications associated with nasopharyngeal stents have been reported previously and include exaggerated swallowing, partial stent obstruction with fur, stent obstruction with tissue, and persistent nasal discharge [1]. The dogs described in this report developed full thickness palatal defects several months following nasopharyngeal stent placement. To the authors’ knowledge, this complication has not been previously reported.

## Case presentation #1

A 3-year-old spayed female Chihuahua weighing 3.4-kg (7.5-lb) was examined at the Veterinary Medical Teaching Hospital (VMTH) at Texas A&M University in February 2012 for sneezing and nasal discharge, described as brown and mucoid. Clinical signs began approximately 4 months prior to presentation and were variably responsive to antimicrobial therapy prescribed by the referring veterinarian, including cefovicin, enrofloxacin, amoxicillin-clavulanic acid, and sulfadimethoxine-ormetoprim. Diphenhydramine and prednisone were also administered without clinical improvement. In the two weeks prior to this visit, the discharge had increased substantially and the dog’s appetite had decreased.

**Figure 1:**
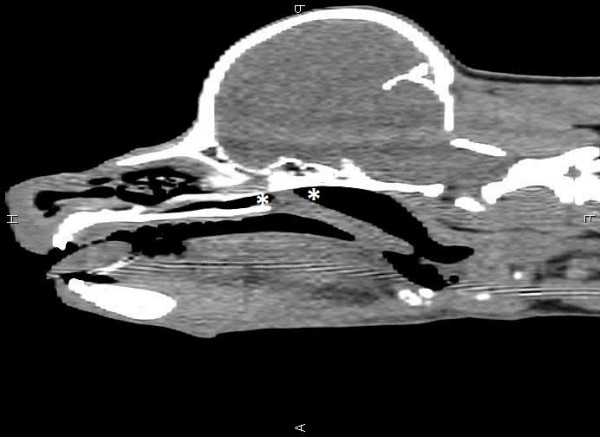
**Sagittal CT image of the head of Dog 1.** The extent of the nasopharyngeal stenosis is marked by two white asterisks.

**Figure 2:**
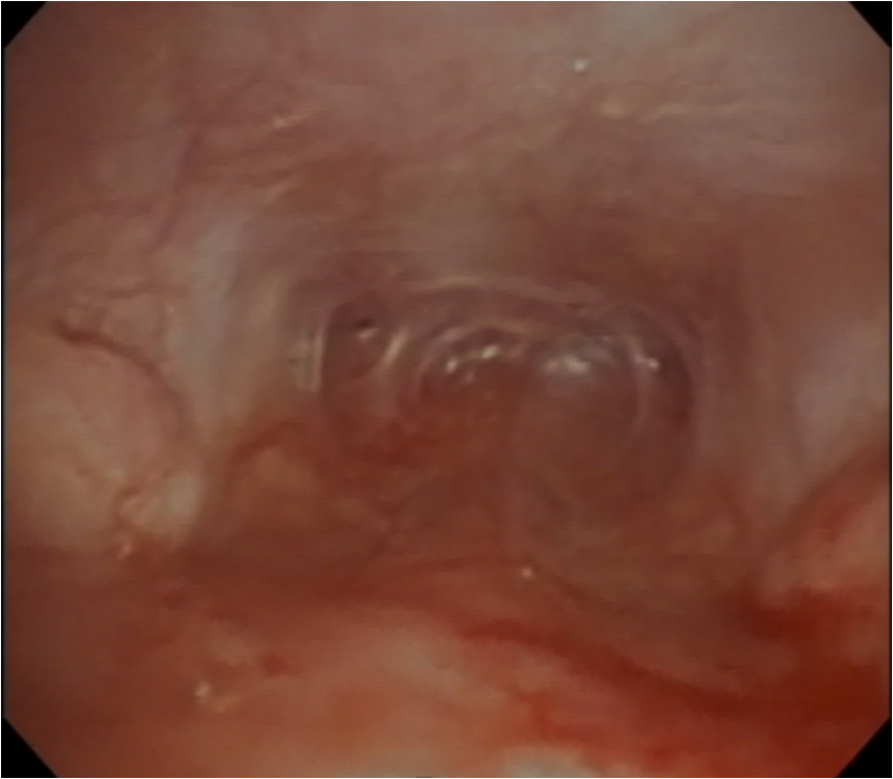
Retroflexed endoscopic view of the nasopharyngeal area in Dog 1 prior to stent placement.

The patient was initially referred to the VMTH in April 2010 for stertorous breathing following routine ovariohysterectomy. At that time, the dog was diagnosed with nasopharyngeal stenosis based on retroflexed endoscopic examination, presumed to be the result of regurgitation of gastric contents whilst under anesthesia. A small opening (1–2 mm) was present in the center of the affected area. At that visit, the stenotic area was dilated with a balloon catheter placed over a guidewire using endoscopic guidance. Prednisolone (0.44 mg/kg [0.2 mg/lb] PO q 12 hrs for 7 days, then q 24 hrs) and omeprazole (0.6 mg/kg [0.27 mg/lb] PO q 24 hrs for 7 days) were administered post-operatively. Transient improvement was noted, but the stertorous breathing recurred within 7 days. Computed tomography (CT), performed 2 weeks after ballooning, indicated a soft tissue density in the rostral nasopharynx caudal to the hard palate causing near complete occlusion of the airway in that location (Figure 1). The length and diameter of the affected area as well as the diameter of the nasopharynx rostral and caudal to the stenosis were determined from sagittal and axial CT images as previously described [1] and a covered 10 mm × 22 mm balloon expandable metal stent^a^ was ordered. The dog returned to the VMTH in June 2010 and was placed under general anesthesia in left lateral recumbency for stent placement. The choanal area was visualized with a retroflexed bronchoscope and complete stenosis was evident (Figure 2). It was assumed that the prior ballooning procedure had triggered complete closure of the affected area. A flexible insertion sheath^b^ was advanced caudally through the left nostril, until it contacted the stenotic area. Appropriate dorsoventral orientation was verified fluoroscopically. A 21 gauge, 13 mm needle^c^ was inserted through the flexible sheath and advanced through the stenotic tissue until visualized endoscopically in the choanal area. A 0.025” guidewire was placed through the needle and across the stenotic area; the needle and sheath were then removed. The affected area was dilated initially with an 8 mm × 10 mm balloon catheter which was exchanged for a 10 mm × 10 mm balloon catheter for repeated inflations. The balloon catheter was then removed and the stent was placed under fluoroscopic guidance across the stenotic area and inflated. The distal portion of the stent was viewed endoscopically during deployment. Post-operative therapy included tramadol 3 mg/kg [1.4 mg/lb] PO q 8 hrs for 7 days), amoxicillin-clavulanate (15 mg/kg [6.8 mg/lb] PO q 12 hrs for 10 days) and prednisolone (0.44 mg/kg [0.2 mg/lb] PO q 12 hrs for 14 days). The patient improved immediately following stent placement and was breathing comfortably when discharged the following day.

When examined in February 2012, the dog was quiet and reserved, with a body condition score of 4/9. Rectal temperature was 100.9°F, pulse was 140 beats/min and respiratory rate was 24 breaths/min. A grade II/VI left-sided systolic murmur was noted; there were no arrhythmias or pulse deficits. The remainder of the physical examination within normal limits. There was a strong odor noted, emanating from the dog’s nasal area and mouth. Purulent discharge was noted around both nares. Airflow was assessed using a glass slide and appeared to be decreased through both nasal passages. The dog would not permit examination of the oral cavity.

Results of a complete blood count and serum electrolyte panel were within normal limits. A three view thoracic radiographic study was within normal limits. A small volume of fluid was noted in the caudal thoracic esophagus.

**Figure 3:**
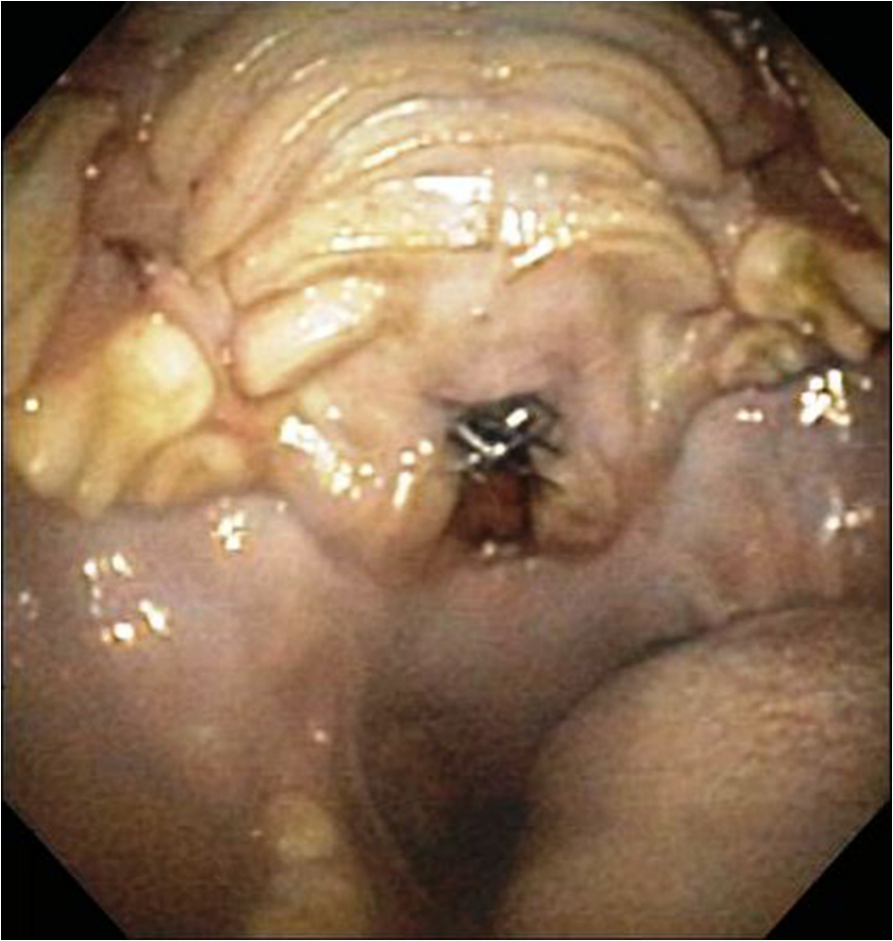
**Image of the palate and oropharynx of Dog 1.** A defect is evident in the caudal palate, with a section of stent visible within the defect.

**Figure 4:**
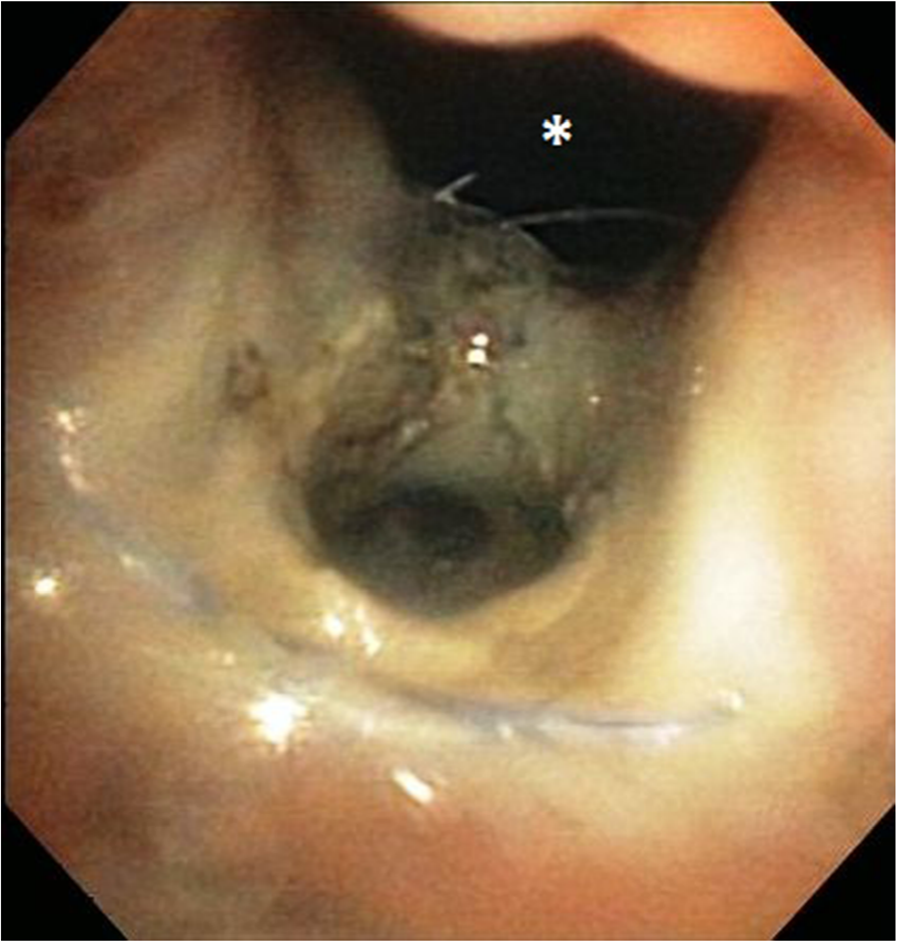
**Retroflexed endoscopic image of the nasopharynx of Dog 1.** The metallic stent is visible in the center of the image and the oronasal fistula is visible at the top of the image (asterisk).

General anesthesia was induced the following day, and the dog was placed in sternal recumbency. When the mouth was opened, a large palatal defect was noted, approximately 5 mm wide and 8 mm long, bridging the transition from hard to soft palate. The caudal portion of the metal stent was visible within the tissue defect, bulging ventrally into the oral cavity (Figure 3). Copious amounts of purulent material and necrotic tissue were associated with the defect. Retroflexed examination of the nasopharynx confirmed that the distal end of the stent had eroded ventrally into the oral cavity (Figure 4).

Aerobic culture of the exudate was performed, and a mixed bacterial population was cultured, including an extended spectrum beta lactamase positive *Escherichia coli*, *Pseudomonas aeruginosa* and an alpha hemolytic *Streptococcus* species. Surgical options, including removal of the stent and closure of the palate were discussed with the owner, but declined. The dog was euthanized 8 weeks later.

## Case presentation #2

A 2-year-old spayed female Chihuahua weighing 2.8-kg (6.2-lb) was examined at the Small Animal Hospital at University of Florida in July 2011 for persistent nasal discharge and open mouth breathing. The patient underwent routine ovariohysterectomy in June 2010; regurgitation of gastric contents was noted during the procedure. Bilateral nasal discharge and stertorous breathing began soon after surgery. The dog was initially treated with a variety of antibiotics with variable response. CT performed in November 2010 revealed nasopharyngeal stenosis, with concurrent rhinitis and turbinate lysis. A dorsal rhinotomy with complete turbinectomy was performed in December 2010. In February 2011, the dog was referred to University of Florida. CT, rhinoscopy and positive contrast rhinography were performed and confirmed nasopharyngeal obstruction. An osseous component to the nasopharyngeal stenosis was present with only 1.1 mm of distance between the palatine bone and the sphenoid bone. The nasal septum, rostral and mid-cavity turbinates were absent, consistent with the previous surgery. Because of the bony abnormality associated with the nasopharyngeal stenosis, a ventral rhinotomy was elected. The dog underwent general anesthesia and pharyngeal intubation. The rhinotomy was performed through a midline incision. The incision extended through the caudal hard palate and into the rostral soft palate, leaving the caudal aspect of the soft palate intact. A pneumatic burr was used to remove a window in the caudal aspect of the palatine bone. Purulent material was present in the choana and was removed. Soft tissue was removed with a combination of sharp and blunt dissection. The pneumatic burr was used to create a trough through the sphenoid bone. The trough was gradually enlarged until an 18 French red rubber catheter could be placed through the bony trough and choana. The red rubber catheter was advanced to extend through the stenotic area to the right nostril, and was sutured flush to the skin to serve as a temporary nasopharyngeal stent. The nasal mucosa was closed with 5–0 polydioxanone in a simple continuous pattern and the oral mucoperiosteum was closed with simple continuous pattern, using 5–0 polydioxanone. Immediate postoperative therapy included methadone (0.2 mg/kg [0.9 mg/lb] IV every 6 hours) and cefazolin (22 mg/kg [10 mg/lb] IV every 8 hours). The dog was discharged from the hospital with tramadol (2.1 mg/kg [1 mg/lb] PO every 8 hours for 5 days), and cephalexin (30 mg/kg [13.6 mg/lb] PO every 12 hours for 14 days).

A 8 mm × 30 mm covered self-expanding metallic stent^a^ was ordered. Stent diameter was determined from the size of the red rubber catheter (i.e. 6 mm) plus an additional 2 mm as previously described [1]. The stent diameter was determined from the red rubber catheter because the sphenoid bone was modified during surgery and the CT was performed prior to surgery. Stent length was calculated from CT images. The dog returned 6 weeks later and was placed under general anesthesia for stent placement. A guidewire was inserted retrograde through the red rubber catheter. The sutures were removed from the red rubber catheter and it was removed. A balloon catheter was advanced over the guidewire, into the nasopharynx and inflated to 8 mm wide. The balloon was removed and the stent was positioned and deployed using fluoroscopic guidance. The dog improved immediately postoperatively. The dog was administered prednisone (0.44 mg/kg [0.2 mg/lb] PO daily for three days then every other day for 5 days), cephalexin (30 mg/kg [13.6 mg/lb] PO twice daily for 14 days), and colchicine (0.035 mg/kg [0.016 mg/lb] PO daily for 14 days). Colchicine was administered to in an attempt to prevent the formation of granulation tissue at the ends of the stent. The dog was discharged from the hospital with a recommendation to perform nebulization for 10 minutes duration 3–4 times daily with a commercial nebulizer.

**Figure 5:**
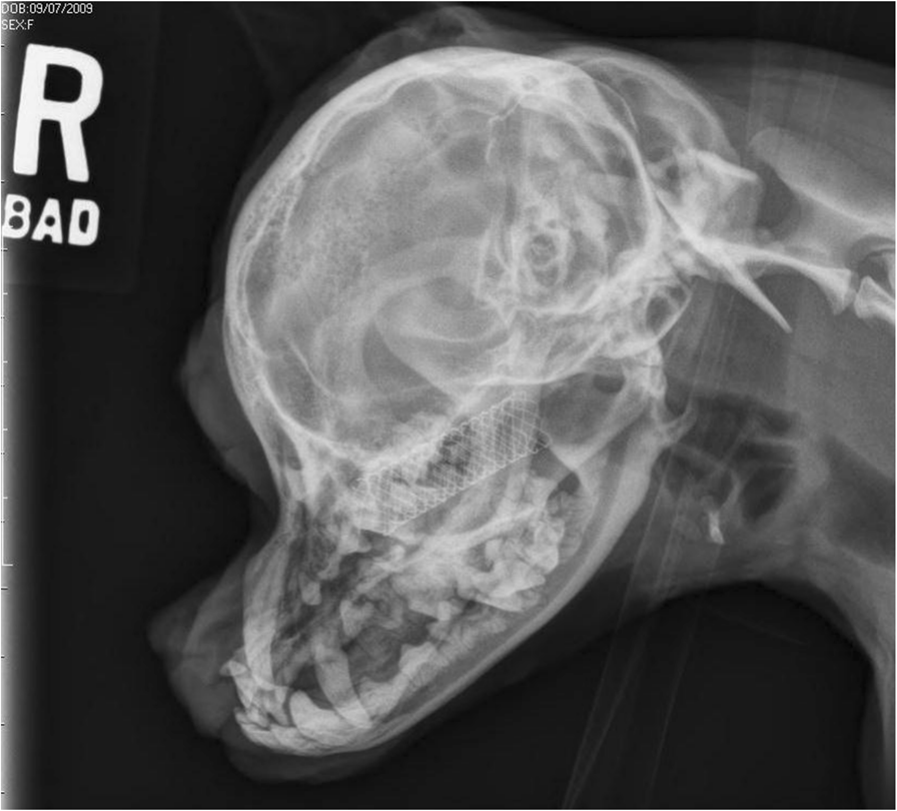
Right lateral radiograph of the skull of Dog 2, taken 3 months after stent placement.

The patient presented for recheck examinations at one and three months following stent placement. At the one month recheck examination, the dog was moving air through both nostrils. The dog continued to experience purulent nasal discharge, but to a much lesser degree than prior to stent placement. Occasionally, the dog pawed at the mouth. Radiographs were performed and showed no movement of the stent (Figure 5).

**Figure 6:**
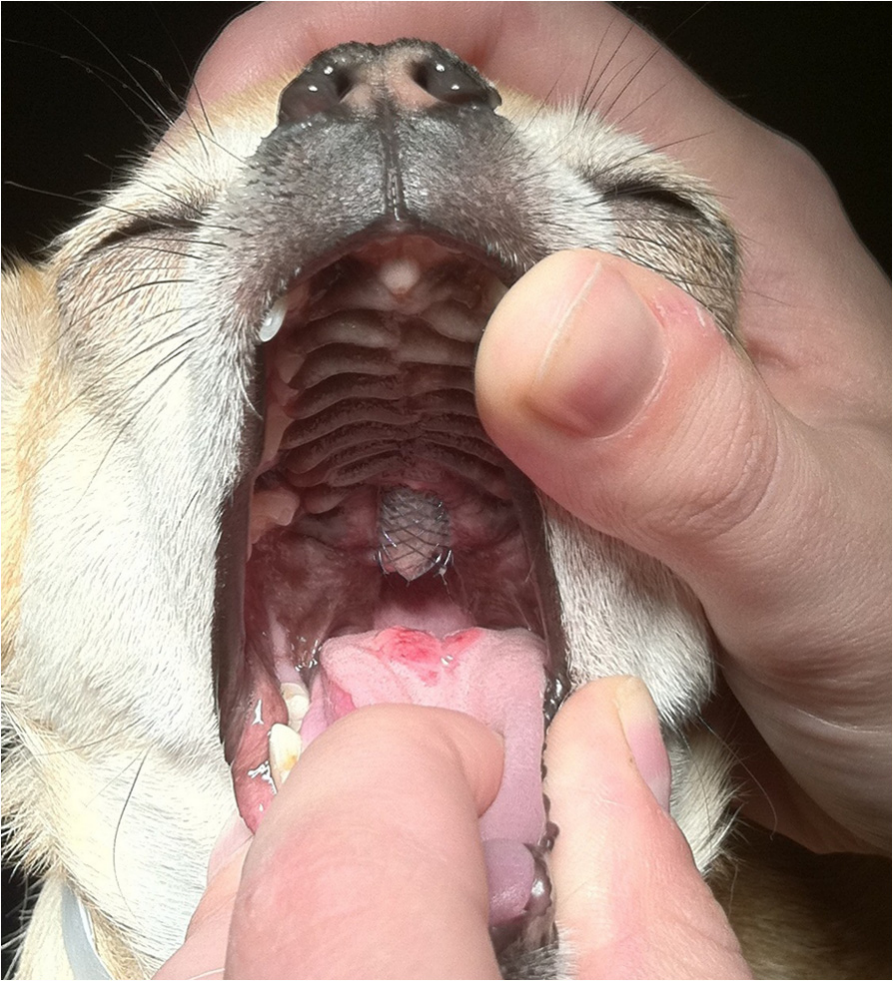
**View of the oral cavity and palate of Dog 2, 4 months following stent placement.** The caudal end of the stent has eroded through the palate. An ulcerated lesion is visible on the tongue.

The dog re-presented for evaluation four months following stent placement. The owners reported severe halitosis, an increased volume of purulent nasal discharge and reluctance to eat. On physical examination, an approximately 7 mm × 7 mm palatal defect was present and a portion of the stent was visible (Figure 6). The dog had ulcerations on the tongue directly below the exposed stent. The ends of the stent were fractured, and some portions of the metal frame were absent. The dog underwent general anesthesia and the stent was removed by grasping the exposed portion and placing gentle caudal and ventral traction. Closure of the oronasal fistula was not attempted.

Nearly one year after removal of the stent, the dog is still able to breathe through the nose. Intermittent mucopurulent nasal discharge and nasal congestion are reported. The owners must hand feed the dog to prevent impaction of food in the oronasal fistula, in part due to the dog’s rapid eating style.

## Conclusions

The authors are not aware of any prior reports describing this complication to stent placement in dogs with acquired nasopharyngeal stenosis. The reason for erosion through the palate is unclear and a direct causal relationship to the metal stent can only be inferred. It is likely however that the stent played a role in development of the palatal defect.

In the first dog described, a guarded needle was used to establish an opening through the stenotic area prior to stent placement. The device was guided fluoroscopically, and appeared appropriately positioned. However, the needle may have been driven through the tissue slightly more ventrally than intended or at an angle, thereby damaging the palate. In the second dog reported here, the palate may have been compromised by the previous surgery, ultimately resulting in pressure necrosis through the tissue. In this dog, it was deemed necessary to perform a ventral rhinotomy to modify the bony abnormalities and provide space for the permanent stent. The ventral rhinotomy procedure and permanent stent placement were staged by six weeks but that interval may not have been long enough for complete healing and establishment of healthy tissue. The two procedures were staged to allow healing of the palate prior to placement of the permanent stent, as the permanent stent would place outward radial force onto the incision site possibly causing dehiscence.

Both covered and uncovered nasopharyngeal stents have been described in veterinary patients. Covered stents are recommended in patients with complete stenosis, as closure has been reported following the use of open stents in this patient population [1]. Covered stents were placed in both dogs in this report, and it is possible that this choice contributed to palatal erosion. Underlying tissue is probably more vulnerable to infection or other compromise underneath a covered stent and therefore more likely to breakdown. A self-expanding stent was placed in the second dog reported here. This was chosen rather than a balloon-expanding stent because a balloon-expanding, covered stent was not available in the size required.

Stent fracture has been reported in dogs with nitinol tracheal stents, and is associated with inflammation of the adjacent tissues [6]. The aboral end of the stent in dog 2 appears to have fractured, and probably contributed to the lesions on the tongue. It is unclear if the stent fractured prior to palatal erosion, or subsequent to that event. Stent fracture would certainly increase the risk of damage to adjacent tissue and may have contributed to the palatine defect. The causes for stent fracture or fraying of the ends of a stent are unclear, but excessive movement or deformity may play a role.

It is interesting to note that both patients reported here were small Chihuahuas. Because of technical limitations, stents may be somewhat oversized in toy breeds if a covered device is required. In small dogs, it is more likely for a substantial portion of the stent to extend into the nasopharynx overlying the soft palate. This tissue is probably subject to substantial compressive forces during swallowing, which may contribute to structural failure of the stent.

Additionally, the skull of a Chihuahua has a unique shape. In dog 2, bony abnormalities were evident on CT examination and at surgery. Such bony restrictions may force the stent to expand only in the ventral direction placing excessive pressure on the palate. There may also be increased mobility of the distal end if the stent is moderately pinched in the stenotic region and therefore unable to expand appropriately.

Erosion of tissue has been reported with stent placement in the respiratory and gastrointestinal tracts in human patients and may be related to stent integration into surrounding tissues [7-9]. Recommendations typically include stent removal if possible, antibiotic therapy and surgical repair of the damaged tissue.

Dog 2 experienced improvement of clinical signs despite the complications associated with the nasopharyngeal stent. The oronasal fistula that was formed by the stent was not addressed surgically and did not close with time despite stent removal. The fistula allowed drainage of nasal mucous into the mouth. Additionally, the dog was able to move air through the nares and bypass the stenotic area. No further rhinogram or rhinoscopy were performed, so it is unknown if the dog moves air thorough the choana, the ornasal fistula or both.

Although nasopharyngeal stenting using permanent expandable metal stents may be an effective solution for patients with nasopharyngeal stenosis, palatal erosion with oronasal fistulation is a possible complication and should be included in any discussion about stent placement.

## Endnotes

^a^Infiniti Medical LLC, Menlo Park, CA.

^b^Olympus America Inc, Center Valley, PA.

^c^Olympus America Inc, Center Valley, PA.

## Competing interests

The authors declare that they have no competing interests.

## Authors’ contributions

AKC participated in stent placement for Dog 1 and identified the fistula. KTM participated in the initial surgery and stent placement for Dog 2 and identified the fistula. ABS participated in stent placement for Dog 1. CEW identified the fistula in Dog 1. LCC participated in in the initial surgery stent placement for Dog 2. GWE participated in in the initial surgery stent placement for Dog 2 and identified the fistula. All authors read and approved the final manuscript.

## Author’s information

Dr. Mankin’s present address is Department of Small Animal Clinical Sciences, College of Veterinary Medicine and Biomedical Sciences, Texas A&M University, College Station, TX 77843.
